# Integrating herbarium specimen observations into global phenology data systems

**DOI:** 10.1002/aps3.1231

**Published:** 2019-03-07

**Authors:** Laura Brenskelle, Brian J. Stucky, John Deck, Ramona Walls, Rob P. Guralnick

**Affiliations:** ^1^ Florida Museum of Natural History University of Florida Gainesville Florida USA; ^2^ Berkeley Natural History Museums University of California Berkeley California USA; ^3^ CyVerse Bio5 Institute The University of Arizona Tucson Arizona USA

**Keywords:** data integration, herbarium specimens, knowledge representation, ontology, plant phenology

## Abstract

**Premise of the Study:**

The Plant Phenology Ontology (PPO) was originally developed to integrate phenology observations of whole plants across different global observation networks. Here we describe a new release of the PPO and associated data pipelines that supports integration of phenology observations from herbarium specimens, which provide historical and modern phenology data.

**Methods and Results:**

Critical changes to the PPO include key terms that describe how measurements from parts of plants, which are captured in most imaged herbarium specimens, relate to whole plants. We provide proof of concept for ingesting annotations from imaged herbarium sheets of *Prunus serotina*, the common black cherry. We then provide an example analysis of changes in flowering timing over the past 125 years, demonstrating the value of integrating herbarium and observational phenology data sets.

**Conclusions:**

These conceptual and technical advances will support the addition of phenology data from herbaria, but also could be expanded upon to facilitate the inclusion of data from photograph‐based citizen science platforms. With the incorporation of herbarium phenology data, new historical baseline data will strengthen the capability to monitor, model, and forecast plant phenology changes.

Plant phenology—the timing of plant life‐cycle events such as flowering or leafing out—plays a fundamental role in the functioning of terrestrial ecosystems, including human agricultural systems (Reilly et al., 1996; Chmielewski et al., 2004; Visser and Both, 2005; Franks et al., 2007; Bertin, 2008; Willis et al., 2008; Miller‐Rushing et al., 2010; Anderson et al., 2012; McKinney et al., 2012; Miller‐Struttmann et al., 2015). Phenology shifts over time are often the most immediate and visible ecological response to environmental change, and as a result, can serve as a “canary in the coal mine” for more drastic ecosystem changes (Parmesan and Yohe, 2003; Menzel et al., 2006; Cleland et al., 2007; Intergovernmental Panel on Climate Change, 2007; Wolkovich et al., 2012; Chuine and Régnière, 2017). Given the need to understand how phenology is changing in response to human pressure, monitoring programs have been set up at regional and continental scales to provide the evidential basis for detecting change (Koch et al., 2010; Rosemartin et al., 2014; Elmendorf et al., 2016; Templ et al., 2018). However, as noted recently (Kissling et al., 2018; Stucky et al., 2018), such systems often have different reporting standards and procedures, which can make broader‐scale interoperability a challenge. Additionally, there are many untapped sources of phenological data over longer time scales, such as herbarium specimens, that could be integrated with data from observation networks, but there are technical challenges to doing so properly.

Initial development of the Plant Phenology Ontology (PPO; Stucky et al., 2018) and associated informatics pipelines (see https://github.com/biocodellc/ppo-data-pipeline and https://github.com/biocodellc/ontology-data-pipeline) have made it possible to integrate heterogeneous plant phenological data from different global observation networks. Using these tools, an integrated phenology knowledge base has been developed that consolidates data from the National Phenology Network (NPN, Rosemartin et al., 2014; https://www.usanpn.org/), the National Ecological Observatory Network (NEON, Elmendorf et al., 2016; http://data.neonscience.org/), and the Pan‐European Phenology Database (PEP725, Koch et al., 2010; Templ et al., 2018; http://www.pep725.eu/). Data in this knowledge base can be accessed via a web portal (http://www.plantphenology.org) and an R package (https://cran.r-project.org/web/packages/rppo/). Although this knowledge base greatly expands the spatial coverage of integrated plant phenological data for research, most phenology observation networks were established within the current century and thus lack historical reports of phenology for most plant taxa (Stucky et al., 2018). Herbarium specimens contain a wealth of historical phenological information that, when annotated and shared, could improve both the temporal and spatial coverage of available phenological data (Davis et al., 2015). With large‐scale imaging of plant specimens as part of national digitization efforts such as iDigBio (Page et al., 2015; http://www.idigbio.org/), there is an unparalleled opportunity to assemble hundreds of millions of new phenology observations based on these imaged herbarium specimens. A key next step in phenological data integration efforts is to integrate phenological data from herbarium specimens with phenological data from field observations.

In this paper, we provide a new framework for integrating herbarium and field phenology observations, and we show that such integration is technically feasible. First, we describe the changes to the PPO needed to integrate phenological data from herbarium records with those from in‐situ observations of whole plants. Then, we describe the updated pipeline tools that take raw, input phenology observations and convert them to a knowledge base of interoperable data available to any interested user. Finally, we provide an example analysis using integrated herbarium and observation data to demonstrate that the increased temporal coverage is useful for documenting phenology change dynamics. We close by briefly discussing potential applications of this work beyond herbarium records, including the burgeoning photographic evidence of phenology coming from incidentally collected citizen science efforts.

## MATERIALS AND METHODS

### Modeling phenology observations from herbarium specimens in the PPO

The PPO provides the standardized terminology, definitions, and logical axioms that are needed for large‐scale phenological data integration. Because observation network data are based on whole plants, and herbarium sheets often do not contain whole plants, our goal was to extend the PPO and the supporting data integration pipeline to enable accurate inferences about phenological data from herbarium specimens.

The first step in accomplishing this was to develop a way to model phenology observations of parts of plants in the logical framework of the PPO. Given its initial design for observation network data, the logical axioms in the PPO connected all phenology observations to stances of the class *‘whole plants’* (from the Plant Ontology; Cooper et al., 2013). To make inferences about herbarium data, we needed a way to relate parts of plants to whole plants, as well as a way to logically translate what a phenology observation of a part of a plant means in the context of a whole plant. To accomplish this, we created a new class in the PPO called *‘portion of a plant’*, which is defined as a *‘plant structure’* (from the Plant Ontology) that ‘*is or was part of’* a *‘whole plant’* (Cooper et al., 2013). (Note that *‘is or was part of’* is also a new object property in the PPO.) To facilitate translation of phenology observations of a *‘portion of a plant’* to information about the associated *‘whole plant’*, we created a new object property called *‘generated from’* to describe the relationship between the original phenology observation data for a *‘portion of a plant’* and another set of phenological data for the *‘whole plant’* of which that *‘portion of a plant’* was a part. With these new entities, we extended the PPO to allow for phenology observations of instances of *‘portion of a plant’* as well as instances of *‘whole plant’*. This new model maintains the PPO logical backbone that relies on instances of whole plants to make inferences without losing accuracy when observing plant parts. The resulting full model for phenology observations of parts of plants is described in detail below (see Results). The addition of new terms and properties to the PPO had nontrivial cascading effects on the existing ontology structure; in all, we had to change axioms for well over 100 terms in the PPO. After finalizing the new version of the PPO, we merged all changes into the main Plant Phenology Ontology repository on GitHub (https://github.com/PlantPhenoOntology/ppo) and created a new ontology release (https://github.com/PlantPhenoOntology/ppo/releases/tag/v2019-01-16).

### Assembling a test data set, formatting and mapping the test data set to the PPO

To provide a herbarium phenology test case for the PPO and our integration pipeline, we generated first‐order phenology scorings (Yost et al., 2018) for images of *P. serotina* Ehrh. We analyzed all images on iDigBio that were linked to digitized specimen records with georeferences. The institutions that house these specimens are listed in Appendix 1.

One of us (R.P.G.) with experience in annotating *Prunus* L. species scored each image for presence or absence of unopened flowers, opened flowers, senesced flowers, and fruits. During scoring, any potential species misidentifications were noted and eliminated from the final data set, because it can be challenging to distinguish *P. serotina* from other *Prunus* species, especially *P. virginiana* L. Scoring of opened flowers, unopened flowers, and fruits followed reporting standards from the NPN (Rosemartin et al., 2014). Transitional cases where early fruits are barely visible but flower material is still present were coded as senesced flowers. These were all later double‐checked by R.P.G., after also scoring multiple other *Prunus* species, in order to verify accuracy. In total, 570 images were scored.

### Changes to the supporting integration pipeline and web portal user interfaces

The phenology data integration pipeline was originally developed for processing whole plant observation data acquired in the field. We extended the pipeline by implementing new rules, detailed in Table 1, for translating observations of a part of a plant to data about the corresponding whole plant.

**Table 1:** Observations of a *‘portion of a plant’* generate an output trait called a *‘data item’*, but these need to be translated into descriptions of phenological traits for the associated *‘whole plant’*. Below is the logical mapping used by the pipeline to make those translations. For example, if an observer reports a lower count of five flowers and upper count of 10 on a herbarium specimen image of a *‘portion of a plant’*, the qualitative reporting is *‘present’* for *‘portion of a plant’*. The mapping for the whole plant output is a lower count of five and an undefined upper count, because it is impossible to know how many flowers were actually on the whole plant.^a^

*‘portion of a plant’* observation data		Output data for the *‘whole plant’*	
Qualitative	Quantitative	Qualitative	Quantitative
Absent	‘lower count’ == 0, ‘upper count’ == 0	Undefined	‘lower count’ = undefined; ‘upper count’ = undefined
Present	‘lower count’ > 0, ‘upper count’ ≥ ‘lower count’	Present	‘lower count’ is the same as for the ‘portion of a plant’; ‘upper count’ = undefined

a Double equal sign signifies “equals,” whereas single equal sign signifies “is.”

The informatics pipeline takes incoming data as comma‐separated value (CSV) files, converts the data to Resource Description Framework (RDF) triples, runs inferencing on the RDF triples, and writes the output data back to a CSV format. Because analyses of phenology observing process data are based on whole plants, and not portions of plants, for now the pipeline only produces output data about whole plants, not portions of plants. All pipeline code is available at: https://github.com/biocodellc/ontology-data-pipeline. The pipeline configurations used for this work can be found at: https://github.com/biocodellc/ppo-data-pipeline.

### Example data analysis

Prior to herbarium data being added to the Global Plant Phenology Portal (http://www.plantphenology.org), the earliest records of opened flowers available for our exemplar species, *P. serotina*, dated to 2007. After we added the herbarium records, we had a total of 969 observations of opened flowers for *P. serotina* dating back to 1875, with 203 herbarium specimen annotations and 766 observations from the NPN.

To analyze the effects of latitude and time on the earliest day of flowering, we fit a multiple linear regression model with *day_of_earliest_flowering* as the response and *year*,* latitude*, and the interaction between *year* and *latitude* as predictors (i.e., *day_of_earliest_flowering* = *year* + *latitude* + latitude*year). To check for biases in observation dates between the two data sources (NPN and herbarium data), we analyzed a linear regression model with *day_of_earliest_flowering* as the response and *latitude* and *data_source* as predictors, using only data from the years 2007–2018, the years for which both NPN and herbarium data were available. Prior to these statistical analyses, we spatially aggregated the data. To do this, we first aggregated all observations to 0.1‐degree grid cells. Then, for each grid cell, the earliest flowering date reported within the grid cell for a given year was the value used for fitting the statistical models. To test for observation biases, these aggregation steps were done separately for each data source.

## RESULTS

### Updated phenology observation model

**Figure 1:**
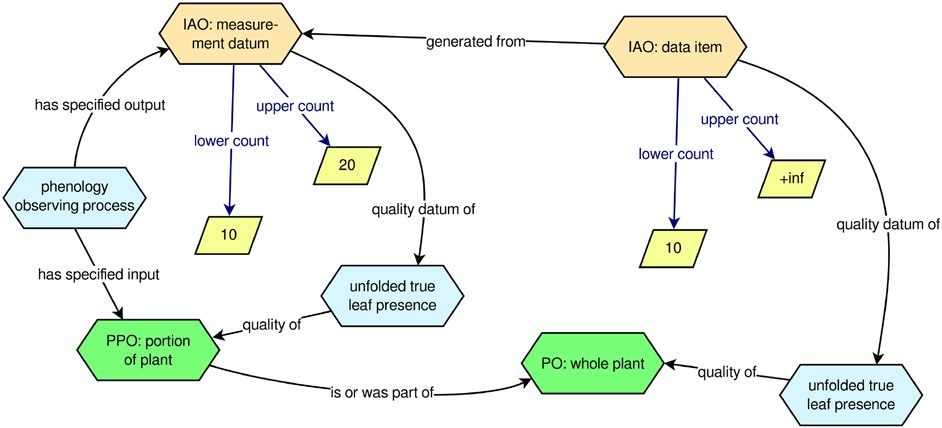
An example of the PPO's model for a phenology observation of a herbarium specimen (or any other *‘portion of a plant’*), on the left, and how that observation can be linked to data about a *‘whole plant’*, on the right. This figure shows the key new class, *‘portion of a plant’*, and three of the new object properties (‘*is or was part of’*,* ‘generated from’*, and *‘quality datum of’*).

The PPO's new model of a phenology observation of a herbarium sheet or any other ‘*portion of a plant*’ (e.g., a citizen science photograph of a branch of tree) is illustrated in Figure 1. In the PPO, *‘portion of a plant’* is a subclass of *‘plant structure’*, similar to the existing term *‘whole plant’*. The overall process model starts with the input of a *‘portion of a plant’* into a *‘phenology observing process’* with an output of a measurement of a *‘plant phenological trait’*. In the example shown in Figure 1, the output of the observing process is a measurement of 10 to 20 *‘unfolded true leaves present’*. In order to make further inferences about whole plants, the relationship *‘is or was part of’* is used to link a *‘portion of a plant’* to a *‘whole plant’*. The term *‘is or was part of’* accounts for the fact that a *‘portion of a plant’* may either be derived from a plant part (i.e., it was at some point part of a whole plant) or may still be part of a plant. Although herbarium specimen images are all of structures that were derived from a plant part, we use “is or was” to be more general and account for future work on images of intact plants that show only part of the plant.

Another key part of the new observation model is the use of *‘generated from’* to link the original *‘measurement datum’*, which is a direct output from the observing process, to an IAO: *‘data item’* (from the Information Artifact Ontology [IAO]; Ceusters, 2012) about a whole plant. We use *‘data item’* because the data about the *‘whole plant’* are derived from the data about the *‘portion of a plant’* and thus are not directly measured (and, therefore, cannot be instances of *‘measurement datum’*). To connect a *‘data item’* to the observed phenological trait, we also minted a new property *‘quality datum of’*, which also has an inverse property *‘has quality datum’* (see Fig. 1). The data about the *‘whole plant’* are generated by the integration pipeline, as discussed above.

We must note that not all herbarium specimens are a *‘portion of a plant’*. Herbarium sheets can and do contain whole plants, and although this is uncommon for *Prunus* and most other woody taxa, it is common for many herbaceous species. The PPO's new data model does not require that herbarium specimens be treated as a *‘portion of a plant’*. Rather, herbarium specimens can also be represented as instances of *‘whole plant’* when appropriate. Even if a herbarium specimen that is a *‘whole plant’* is mistakenly treated as *‘portion of a plant’*, the axioms of the PPO and logic in the pipeline are such that no incorrect inferences will be obtained, although some inferences will be less informative than possible (e.g., observations of absences; see Table 1). There are also cases in which a single herbarium sheet contains multiple specimens; in these cases, a separate observation should be recorded for each specimen to ensure correct reasoning.

### Exemplar data on the Global Plant Phenology Data Portal

The test data set assembled for *P. serotina* was run through the updated PPO data integration pipeline and ultimately added to the Global Plant Phenology knowledge base (http://www.plantphenology.org). The ingest toolkit and all individual steps are described more fully in the plant phenology data pipeline Github repository (https://github.com/biocodellc/ppo-data-pipeline). Mapping files required to perform integration and the ingested data file itself are available on GitHub (https://github.com/biocodellc/ppo-data-pipeline/tree/master/projects/herbarium ), thus providing a general template for further ingestion of new herbarium data.

**Figure 2:**
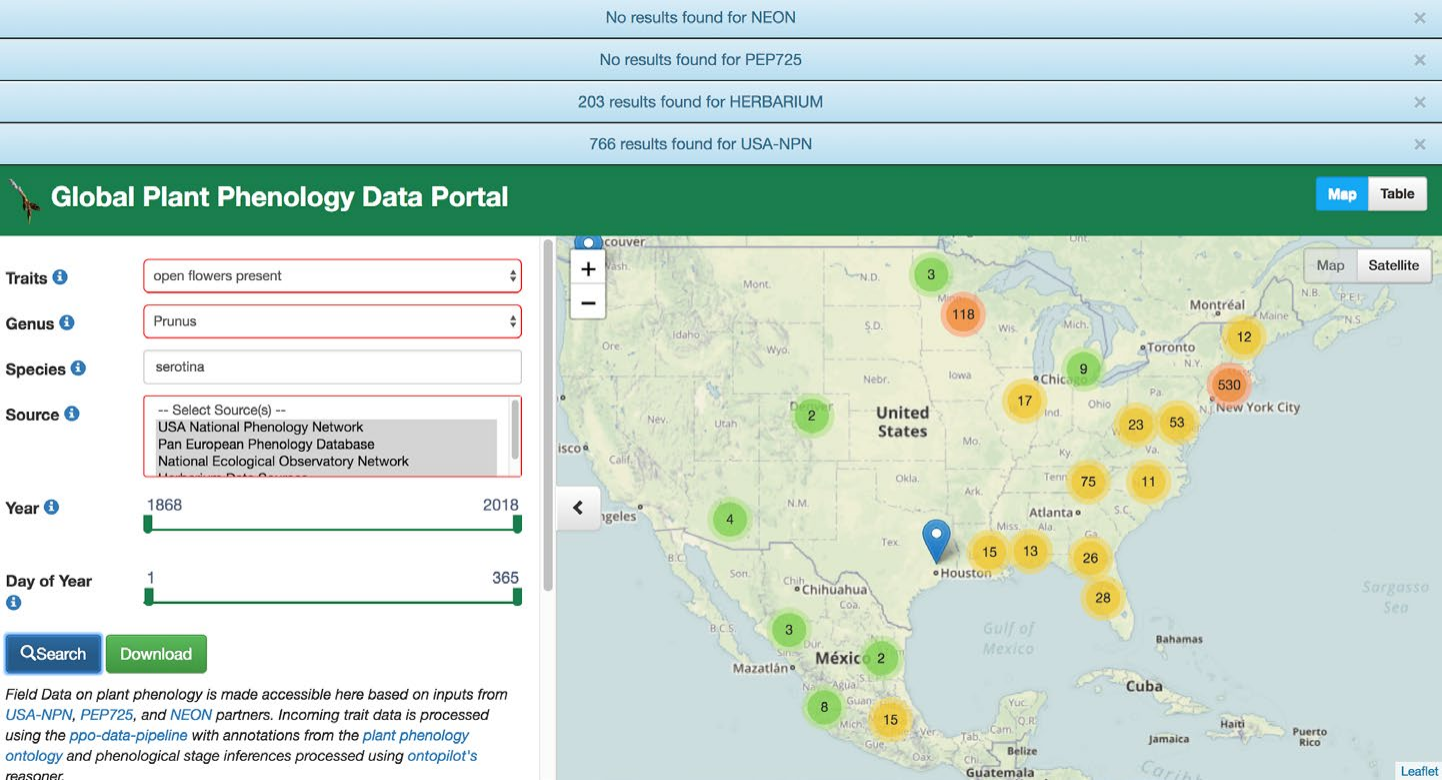
A screenshot of search results for *Prunus serotina* observations of open flowers present on https://www.plantphenology.org. Note the inclusion of record counts for herbarium specimens shown at the top of the figure.

All of these phenological data for *P. serotina* are now available on the online portal, as shown in Figure 2. The portal interface has been adjusted so that at the top of the page, users can see a breakdown of the data sources where these results came from. In the *P. serotina* example, there were 203 total herbarium records with open flowers and 766 open flower observations from the NPN. The page also presents users with two options for how to view these data on the interface via a map visualization (Fig. 2) or a standard table with text fields and values that provide standardized content with the same field headers and other elements for all data resources. All data can be downloaded for further analyses using the “Download” button. All herbarium annotations have Uniform Resource Identifier (URI) links back to the specimen records from which those annotations were made.

### Results of *Prunus serotina* analysis

**Figure 3:**
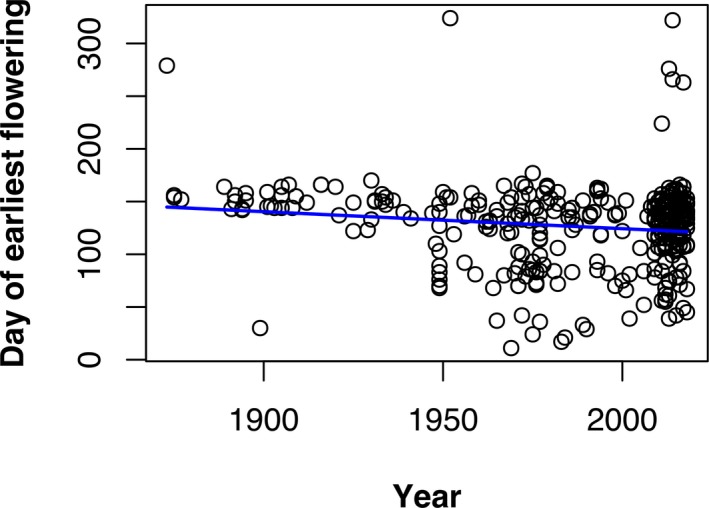
An effect plot showing a trend toward earlier flowering over more than a century, when accounting for latitude.

Our analysis of flowering times revealed that, on average, *P. serotina* in North America has steadily accelerated its flowering times since 1873 (Fig. 3), and this effect was statistically significant after controlling for the effects of latitude (overall model *P* < 0.001, *F* = 105.2 [3 and 366 *df*], adjusted *R* ^2^ = 0.459; see Table 2 for individual coefficient estimates and *P* values). The interaction between *year* and *latitude* was also significant (Table 2), which suggests that phenological shifts vary by geographic location. Our analysis of potential bias in observation dates between the two data sources did not indicate a significant difference in observation dates between NPN and herbarium‐based observations when controlling for latitudinal effects (estimated mean difference between NPN and herbarium‐based observations: 3.13 days, *P* = 0.695). However, the sample sizes for this analysis were extremely unbalanced (NPN: *n* = 178, herbarium: *n* = 16), so it is difficult to draw any definitive conclusions about data source biases.

**Table 2:** A table showing the statistical outputs of the latitude*year model. The overall *P* value for the model was <0.001, and the *R* ^2^ = 0.459

Variable	Coefficient estimate	*P* value
*latitude*	−0.330 days per degree	0.0400
*year*	−0.913 days per year	0.0054
latitude*year	0.0186	0.0218

## DISCUSSION

### Future improvements to the PPO related to portions of plants

We have provided a model and shown proof of concept for assembling data products from herbarium specimens or any other resource that contains parts of plants as opposed to whole plants. To move forward with integrating herbarium‐ and field‐based phenological data, we took some shortcuts in ontology development that allowed us to proceed while we wait for completion of complementary work in external ontologies. Specifically, we created two object properties—*‘is or was part of’* and *‘generated from’*—that are consistent with the logic of the Relations Ontology (RO) (http://purl.obolibrary.org/obo/ro.owl), but can be made more logically meaningful and compatible with other Open Biological and Biomedical Ontology (OBO) Foundry (http://www.obofoundry.org/) ontologies in future releases of the PPO. For “parthood,” we required that some significant portion of the plant is not present or not visible (i.e., stronger than an irreflexive version of the RO: *‘part of’*) both in the present or past tenses. There is currently no easy way to express this using extant terms in OBO ontologies, so our solution is to use the named class *‘portion of a plant’* defined by the *‘is or was part of’* relation and make the inferences of presence traits in the data ingest pipeline, as described above. Because similar kinds of traits exist more widely in biodiversity studies, we will work with the BioCollections Ontology (Walls et al., 2014) to develop a robust ontology design pattern for this kind of data.

The *‘generated from’* relation is a “shortcut” relation that implies an instance of a non‐specified process that takes the observation of a *‘portion of a plant’* as input and generates data about an associated *‘whole plant*’. For the purposes of integrating this initial set of herbarium data, it was not necessary to define this process and specify the full chain of logic leading to the final data. However, implementing the full process model would provide more information and would be more robust to future data needs. Because this pattern of deriving data from an observation is a process that is used widely beyond phenology, rather than implementing it within the PPO, we will wait until the BioCollections Ontology develops the logic for this type of process and then reuse that pattern in the PPO.

### Scaling up with more herbarium specimen data

Large‐scale imaging of natural history collections and associated digitization of specimen label information is well underway with tens of millions of images already available, and with potentially hundreds of millions of images possible. The example analysis with *P. serotina* records showcases the value of integrating herbarium specimen data with more recent, field observation data. Prior to the addition of the 570 phenology observations from herbarium specimens, which include all of the data prior to 2007, an analysis of long‐term temporal change in *P. serotina* phenology would have been challenging. Although this analysis is not meant to determine drivers of change, we did find a significant trend toward earlier flowering over time, when accounting for latitude. Given the millions of herbarium specimen images already available in online databases, there is enormous potential to develop a comprehensive phenology database with a rich historical record that would enable significant new research about phenology changes. Currently, obtaining phenological data from herbarium specimens (or images of specimens) remains a labor‐intensive task, which is a significant barrier for developing large historical phenology data sets. However, crowd‐sourced citizen science platforms such as Notes from Nature (Hill et al., 2012; https://www.notesfromnature.org/) and CrowdCurio (Willis et al., 2017) offer at least a partial solution to this problem. In addition, computer vision techniques could allow for rapid, highly automated extraction of phenological data from specimen images (Lorieul et al., 2019; Stucky et al., unpublished data). Finally, not all herbarium specimens are parts of plants. Sometimes, whole plants are captured on sheets, and there are also cases of multiple plants on a single sheet. Recording this information could allow smarter inferences about how observations of specimens relate to whole plants. This is another area to explore, and potentially also to leverage machine learning approaches.

We close by noting that herbarium specimens are not the only phenology‐relevant resources that feature parts of plants. Novel citizen science platforms are generating a significant volume of plant images, as are other resources such as historical photos or videos that contain phenology‐relevant data. For example, there are currently (as of 24 October 2018) 5860 photographs from 3000 observers of black cherry (*P. serotina*) plants on the citizen science platform iNaturalist (https://www.inaturalist.org). The vast majority of those records were generated in the past two years, suggesting strongly that future growth may be even faster. Full utilization of such resources for phenology represents an untapped potential, and there is no doubt that flowering state can be discerned from most of these opportunistic reports. The innovations here for integrating herbarium specimens will work just as well for phenology trait capture from citizen science photographs of parts of plants. It is via a combination of data sets that we will be best able to examine the longer‐term picture of phenology change, and to put together the most comprehensive data possible to continue monitoring phenology. We argue that data integration from multiple resources using tools such as the PPO and ontology‐informed data integration pipelines is a critical step to meet phenology monitoring and modeling goals.

## AUTHOR CONTRIBUTIONS

L.B., R.P.G., B.J.S., and R.W. conceived of this study. R.P.G. performed data collection for the proof of concept work and L.B., R.P.G., and B.J.S. performed the statistical analyses presented here. B.J.S., L.B., and R.W. made needed changes to the Plant Phenology Ontology and dealt with managing new releases. J.D., with help from L.B., adapted the mapping files and updated the pipeline to support herbarium data integration. L.B., R.P.G., B.J.S., R.W., and J.D. all helped write the paper.

## Data Availability

The original input CSV file for the *Prunus serotina* example data set is archived on Zenodo https://doi.org/10.5281/zenodo.1473702.

## APPENDIX 1 List of source institutions for *Prunus serotina* herbarium data.

**Table:**

Institution	Herbarium name	Index Herbariorum code
Arizona State University	Arizona State University Vascular Plant Herbarium	ASU
Bartlett Arboretum and Gardens	Bartlett Arboretum Herbarium	BART
Boise State University	Snake River Plains Herbarium	SRP
Brown University	Brown University Herbarium	BRU
Central Michigan University	Central Michigan University Herbarium	CMC
College of Idaho	Harold M. Tucker Herbarium	CIC
Denver Botanic Gardens	Kathryn Kalmbach Herbarium of Vascular Plants	KHD
Desert Botanical Garden	Desert Botanical Garden Herbarium	DES
Eastern Kentucky University	Eastern Kentucky University Herbarium	EKY
Eastern Michigan University	Eastern Michigan University Herbarium	EMC
Eastern Washington University	Eastern Washington University Herbarium	EWU
Florida State University	Robert K. Godfrey Herbarium	FSU
Louisiana State University	Shirley C. Tucker Herbarium	LSU
Mississippi State University	Mississippi State University Herbarium	MISSA
North Carolina State University	North Carolina State University Herbarium	NCSC
Portland State University	Portland State University Herbarium	HPSU
The University of Texas at Austin	Plant Resources Center	TEX
United States Fish and Wildlife Service		VFWO
Florida Museum of Natural History	University of Florida Herbarium	FLAS
University of Georgia	University of Georgia Herbarium	GA
University of Idaho	Stillinger Herbarium	ID
University of Mary Washington	University of Mary Washington Herbarium	MWCF
University of Massachusetts Amherst	University of Massachusetts Herbarium Amherst	MASS
University of Michigan	University of Michigan Herbarium	MICH
University of Minnesota	University of Minnesota Herbarium	MIN
University of North Carolina at Chapel Hill	University of North Carolina at Chapel Hill Herbarium	NCU
University of Rhode Island	University of Rhode Island Herbarium	KIRI
University of South Florida	University of South Florida Herbarium	USF
University of Southern Mississippi	University of Southern Mississippi Herbarium	USMS
University of Washington	University of Washington Herbarium	WTU
University of Wisconsin–Madison	Wisconsin State Herbarium	WIS
Western Carolina University	Western Carolina University Herbarium	WCUH
Yale University	Yale University Herbarium	YU

## References

[aps31231-bib-0001] Anderson, J. T. , D. W. Inouye , A. M. McKinney , R. I. Colautti , and T. Mitchell‐Olds . 2012 Phenotypic plasticity and adaptive evolution contribute to advancing flowering phenology in response to climate change. Proceedings of the Royal Society B. Biological Sciences 279: 3843–3852. 10.1098/rspb.2012.1051 PMC3415914 22787021

[aps31231-bib-0002] Bertin, R. I. 2008 Plant phenology and distribution in relation to recent climate change. Journal of the Torrey Botanical Society 135: 126–146.

[aps31231-bib-0003] Ceusters, W. 2012 An information artifact ontology perspective on data collections and associated representational artifacts. Studies in Health Technology and Informatics 180: 68–72. 22874154

[aps31231-bib-0004] Chmielewski, F.‐M. , A. Müller , and E. Bruns . 2004 Climate changes and trends in phenology of fruit trees and field crops in Germany, 1961–2000. Agricultural and Forest Meteorology 121: 69–78.

[aps31231-bib-0005] Chuine, I. , and J. Régnière . 2017 Process‐based models of phenology for plants and animals. Annual Review of Ecology, Evolution, and Systematics 48: 159–182.

[aps31231-bib-0006] Cleland, E. E. , I. Chuine , A. Menzel , H. A. Mooney , and M. D. Schwartz . 2007 Shifting plant phenology in response to global change. Trends in Ecology and Evolution 22: 357–365. 17478009 10.1016/j.tree.2007.04.003

[aps31231-bib-0007] Cooper, L. , R. L. Walls , J. Elser , M. A. Gandolfo , D. W. Stevenson , B. Smith , J. Preece , et al. 2013 The Plant Ontology as a tool for comparative plant anatomy and genomic analyses. Plant and Cell Physiology 54: e1. 23220694 10.1093/pcp/pcs163 PMC3583023

[aps31231-bib-0008] Davis, C. C. , C. G. Willis , B. Connolly , C. Kelly , and A. M. Ellison . 2015 Herbarium records are reliable sources of phenological change driven by climate and provide novel insights into species’ phenological cueing mechanisms. American Journal of Botany 102: 1599–1609. 26451038 10.3732/ajb.1500237

[aps31231-bib-0009] Elmendorf, S. C. , K. D. Jones , B. I. Cook , J. M. Diez , C. A. F. Enquist , R. A. Hufft , M. O. Jones , et al. 2016 The plant phenology monitoring design for the National Ecological Observatory Network. Ecosphere 7: e01303.

[aps31231-bib-0010] Franks, S. J. , S. Sim , and A. E. Weis . 2007 Rapid evolution of flowering time by an annual plant in response to a climate fluctuation. Proceedings of the National Academy of Sciences USA 104: 1278–1282. 10.1073/pnas.0608379104 PMC1783115 17220273

[aps31231-bib-0011] Hill, A. , R. Guralnick , A. Smith , A. Sallans , R. Gillespie , M. Denslow , J. Gross , et al. 2012 The Notes from Nature tool for unlocking biodiversity records from museum records through citizen science. ZooKeys 209: 219–233. 10.3897/zookeys.209.3472 PMC3406478 22859890

[aps31231-bib-0012] Intergovernmental Panel on Climate Change . 2007 Climate Change 2007—The physical science basis: Working Group I contribution to the Fourth Assessment Report of the IPCC. Cambridge University Press, New York, New York, USA.

[aps31231-bib-0013] Kissling, W. D. , J. A. Ahumada , A. Bowser , M. Fernandez , N. Fernández , E. A. García , R. P. Guralnick , et al. 2018 Building essential biodiversity variables (EBVs) of species distribution and abundance at a global scale: Building global EBVs. Biological Reviews 93: 600–625. 28766908 10.1111/brv.12359

[aps31231-bib-0014] Koch, E. , S. Adler , W. Lipa , M. Ungersböck , and S. Zach‐Hermann . 2010 The Pan European Phenological Database PEP725 *In* Matzarakis A., Mayer H., and Chmielewski F.‐M. [eds.], Proceedings of the 7th Conference on Biometeorology, 331–335. Berichte des Meteorologischen Instituts der Albert‐Ludwigs‐Universität Freiburg no. 20. Albert‐Ludwigs‐University, Freiburg, Germany.

[aps31231-bib-0015] Lorieul, T. , K. D. Pearson , E. R. Ellwood , H. Goëau , J.‐F. Molino , P. W. Sweeney , J. M. Yost , et al. 2019 Toward a large‐scale and deep phenological stage annotation of herbarium specimens: Case studies from temperate, tropical, and equatorial floras. Applications in Plant Sciences 7(3): e1233. 10.1002/aps3.1233 PMC6426157 30937225

[aps31231-bib-0016] McKinney, A. M. , P. J. CaraDonna , D. W. Inouye , B. Barr , C. D. Bertelsen , and N. M. Waser . 2012 Asynchronous changes in phenology of migrating broad‐tailed hummingbirds and their early‐season nectar resources. Ecology 93: 1987–1993. 23094369 10.1890/12-0255.1

[aps31231-bib-0017] Menzel, A. , T. H. Sparks , N. Estrella , E. Koch , A. Aasa , R. Ahas , K. Alm‐Kübler , et al. 2006 European phenological response to climate change matches the warming pattern. Global Change Biology 12: 1969–1976.

[aps31231-bib-0018] Miller‐Rushing, A. J. , T. T. Høye , D. W. Inouye , and E. Post . 2010 The effects of phenological mismatches on demography. Philosophical Transactions of the Royal Society of London. Series B, Biological Sciences 365: 3177–3186. 20819811 10.1098/rstb.2010.0148 PMC2981949

[aps31231-bib-0019] Miller‐Struttmann, N. E. , J. C. Geib , J. D. Franklin , P. G. Kevan , R. M. Holdo , D. Ebert‐May , A. M. Lynn , et al. 2015 Functional mismatch in a bumble bee pollination mutualism under climate change. Science 349: 1541–1544. 26404836 10.1126/science.aab0868

[aps31231-bib-0020] Page, L. M. , B. J. MacFadden , J. A. Fortes , P. S. Soltis , and G. Riccardi . 2015 Digitization of biodiversity collections reveals biggest data on biodiversity. Bioscience 65: 841–842.

[aps31231-bib-0021] Parmesan, C. , and G. Yohe . 2003 A globally coherent fingerprint of climate change impacts across natural systems. Nature 421: 37–42. 12511946 10.1038/nature01286

[aps31231-bib-0022] Reilly, J. , W. Baethgen , F. E. Chege , S. C. van de Geijn , L. Erda , A. Iglesias , G. Kenny , et al. 1996 Agriculture in a changing climate: Impacts and adaptation *In* Climate Change 1995; Impacts, adaptations and mitigation of climate change: Scientific‐technical analyses, 427–467. Cambridge University Press, Cambridge, United Kingdom.

[aps31231-bib-0023] Rosemartin, A. H. , T. M. Crimmins , C. A. F. Enquist , K. L. Gerst , J. L. Kellermann , E. E. Posthumus , E. G. Denny , et al. 2014 Organizing phenological data resources to inform natural resource conservation. Biological Conservation 173: 90–97.

[aps31231-bib-0024] Stucky, B. J. , R. Guralnick , J. Deck , E. G. Denny , K. Bolmgren , and R. Walls . 2018 The plant phenology ontology: A new informatics resource for large‐scale integration of plant phenology data. Frontiers in Plant Science 9: 517. 29765382 10.3389/fpls.2018.00517 PMC5938398

[aps31231-bib-0025] Templ, B. , E. Koch , K. Bolmgren , M. Ungersböck , A. Paul , H. Scheifinger , T. Rutishauser , et al. 2018 Pan European Phenological Database (PEP725): A single point of access for European data. International Journal of Biometeorology 62: 1109–1113. 29455297 10.1007/s00484-018-1512-8

[aps31231-bib-0026] Visser, M. E. , and C. Both . 2005 Shifts in phenology due to global climate change: The need for a yardstick. Proceedings of the Royal Society B. Biological Sciences 272: 2561–2569. 10.1098/rspb.2005.3356 PMC1559974 16321776

[aps31231-bib-0027] Walls, R. L. , J. Deck , R. Guralnick , S. Baskauf , R. Beaman , S. Blum , S. Bowers , et al. 2014 Semantics in support of biodiversity knowledge discovery: An introduction to the biological collections ontology and related ontologies. PLoS One 9: e89606. 24595056 10.1371/journal.pone.0089606 PMC3940615

[aps31231-bib-0028] Willis, C. G. , B. Ruhfel , R. B. Primack , A. J. Miller‐Rushing , and C. C. Davis . 2008 Phylogenetic patterns of species loss in Thoreau's woods are driven by climate change. Proceedings of the National Academy of Sciences USA 105: 17029–17033. 10.1073/pnas.0806446105 PMC2573948 18955707

[aps31231-bib-0029] Willis, C. G. , E. Law , A. C. Williams , B. F. Franzone , R. Bernardos , L. Bruno , C. Hopkins , et al. 2017 CrowdCurio: An online crowdsourcing platform to facilitate climate change studies using herbarium specimens. New Phytologist 215: 479–488. 28394023 10.1111/nph.14535

[aps31231-bib-0030] Wolkovich, E. M. , B. I. Cook , J. M. Allen , T. M. Crimmins , J. L. Betancourt , S. E. Travers , S. Pau , et al. 2012 Warming experiments underpredict plant phenological responses to climate change. Nature 485: 494–497. 22622576 10.1038/nature11014

[aps31231-bib-0031] Yost, J. M. , P. W. Sweeney , E. Gilbert , G. Nelson , R. Guralnick , A. S. Gallinat , E. R. Ellwood , et al. 2018 Digitization protocol for scoring reproductive phenology from herbarium specimens of seed plants. Applications in Plant Sciences 6: e1022. 29732253 10.1002/aps3.1022 PMC5851559

